# Supplementary material for: RNA-mediated condensation of TFE3 oncofusions facilitates transcriptional hub formation to promote translocation renal cell carcinoma
Source: Nat Commun. 2025 Sep 30;16:8712. doi: 10.1038/s41467-025-63761-z (PMC12484640; doi:10.1038/s41467-025-63761-z)
Supplement: Supplementary file 1 — Supplementary Information [file 41467_2025_63761_MOESM1_ESM.pdf]

Supplementary information

RNA-mediated condensation of TFE3 oncofusions facilitates transcriptional hub formation to promote translocation renal cell carcinoma

Lei Guo[1,2,*,#], Rongjie Zhao[1,*],Yi-Tsang Lee[1,*], Junhua Huang[1], James Wengler[1], Logan Rivera[1], Tingting Hong[1], Tianlu Wang[1], Kunjal Rathod[1], Ashley Suris [1], Yitian Wu[1], Xiaoli Cai[1], Rui Wang[1], Yubin Zhou[1,2,#], Yun Huang[1,2,3,#],

1. Institute of Biosciences and Technology, Texas A&M University, Houston, TX, USA
2. Department of Translational Medical Sciences, School of Medicine, Texas A&M University, Houston, TX, USA
3. Department of Biomedical Engineering, College of Engineering, Texas A&M University, College Station, TX, USA

* These authors contributed equally
# These authors jointly supervised this work

Correspondence to: guoleijay@tamu.edu, yubinzhou@tamu.edu, yun.huang@tamu.edu

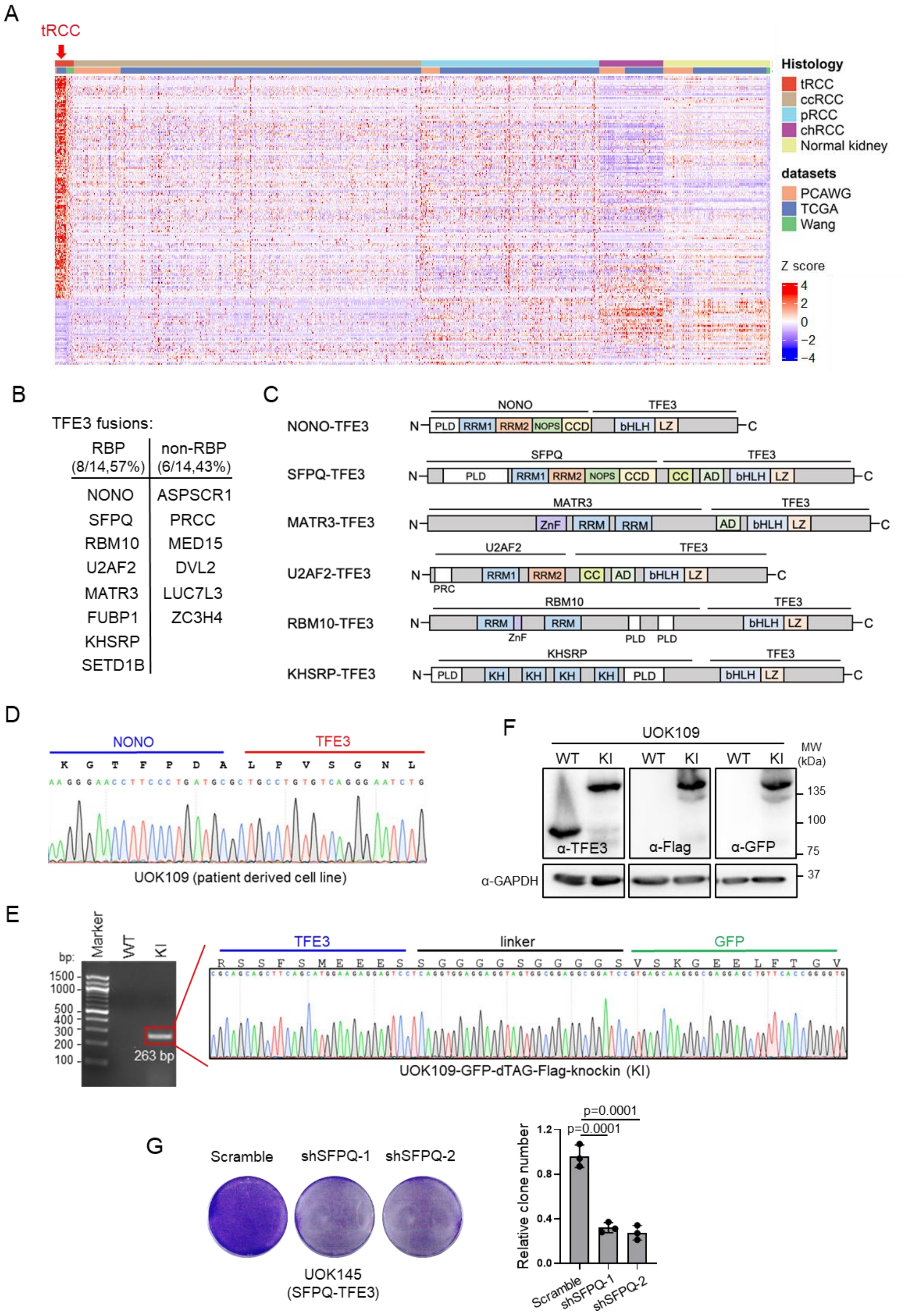

A
tRCC
Histology
tRCC
ccRCC
pRCC
chRCC
Normal kidney
datasets
PCAWG
TCGA
Wang
Z score
4
2
0
−2
−4
B
TFE3 fusions:
RBP (8/14,57%)
non-RBP (6/14,43%)
NONO
SFPQ
RBM10
U2AF2
MATR3
FUBP1
KHSRP
SETD1B
ASPSCR1
PRCC
MED15
DVL2
LUC7L3
ZC3H4
C
NONO-TFE3
SFPQ-TFE3
MATR3-TFE3
U2AF2-TFE3
RBM10-TFE3
KHSRP-TFE3
D
NONO
TFE3
UOK109 (patient derived cell line)
F
UOK109
WT KI WT KI WT KI
MW (kDa)
135
100
75
37
α-TFE3
α-Flag
α-GFP
α-GAPDH
E
Marker
WT
KI
bp:
1500
1000
500
400
300
200
100
263 bp
TFE3
linker
GFP
UOK109-GFP-dTAG-Flag-knockin (KI)
G
Scramble
shSFPQ-1
shSFPQ-2
UOK145 (SFPQ-TFE3)
p=0.0001
p=0.0001
Relative clone number

Supplementary Figure 1. Generating Degron system in patient-derived UOK109 cells. (Related to Figure 1)

(A) Heatmap of genes overexpressed in tRCC as compared with other RCC subtypes or normal kidney across all datasets.

(B) Summary of major TFE3 fusion partners identified from clinical samples. RBP: RNA binding protein.

(C) Scheme showing the domain structures of RBP-fused TFE3 onco-fusions.

(D) Validation of NONO-TFE3 fusions in the patient-derived UOK109 cell line using Sanger's sequencing of the junction site between NONO and TFE3.

(E, F) Validation of NONO-TFE3-GFP-dTAG-Flag expression in the UOK109 knocking-in (UOK109-KI) cell line by Sanger sequencing (E) and immunoblotting (F). The whole cell lysates were subjected to gel electrophoresis and subsequently probed with the indicated antibodies.

(G) Representative images (left) and quantification (right) of the colony formation assay results using UOK145 cells transfected with the scrambled shRNA or two different shRNAs against SFPQ. n = 3 independent biological replicates; one-way ANOVA with Tukey's post-hoc test. Data are shown as mean ± SD. Source data are provided as a Source Data file.

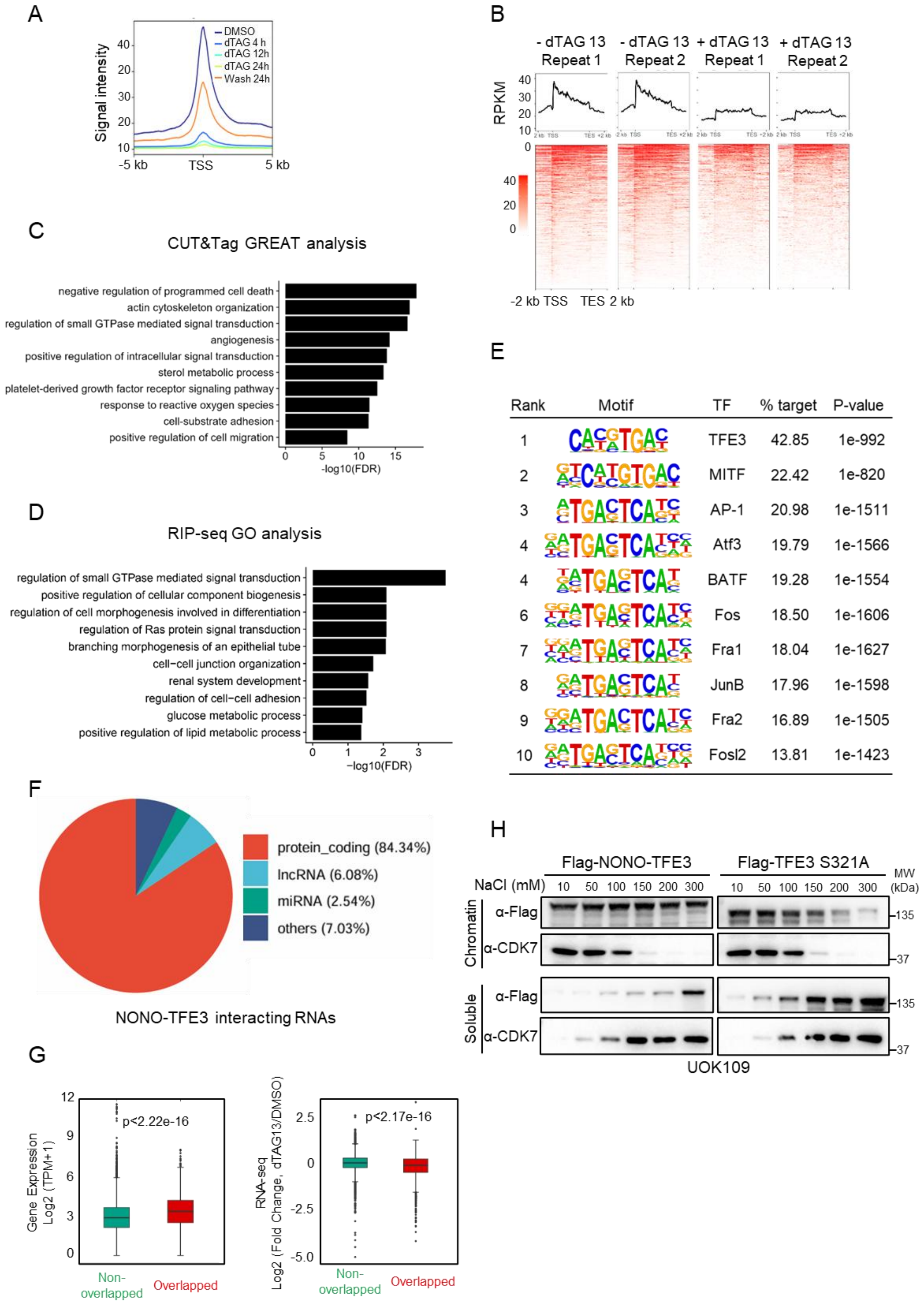
A
Signal intensity
DMSO
dTAG 4 h
dTAG 12h
dTAG 24h
Wash 24h
-5 kb
TSS
5 kb
B
- dTAG 13 Repeat 1
- dTAG 13 Repeat 2
+ dTAG 13 Repeat 1
+ dTAG 13 Repeat 2
RPKM
-2 kb TSS
TES 2 kb
C
CUT&Tag GREAT analysis
negative regulation of programmed cell death
actin cytoskeleton organization
regulation of small GTPase mediated signal transduction
angiogenesis
positive regulation of intracellular signal transduction
sterol metabolic process
platelet-derived growth factor receptor signaling pathway
response to reactive oxygen species
cell-substrate adhesion
positive regulation of cell migration
-log10(FDR)
D
RIP-seq GO analysis
regulation of small GTPase mediated signal transduction
positive regulation of cellular component biogenesis
regulation of cell morphogenesis involved in differentiation
regulation of Ras protein signal transduction
branching morphogenesis of an epithelial tube
cell−cell junction organization
renal system development
regulation of cell−cell adhesion
glucose metabolic process
positive regulation of lipid metabolic process
−log10(FDR)
E
Rank | Motif | TF | % target | P-value
1 | TFE3 | 42.85 | 1e-992
2 | MITF | 22.42 | 1e-820
3 | AP-1 | 20.98 | 1e-1511
4 | Atf3 | 19.79 | 1e-1566
4 | BATF | 19.28 | 1e-1554
6 | Fos | 18.50 | 1e-1606
7 | Fra1 | 18.04 | 1e-1627
8 | JunB | 17.96 | 1e-1598
9 | Fra2 | 16.89 | 1e-1505
10 | Fosl2 | 13.81 | 1e-1423
F
protein_coding (84.34%)
lncRNA (6.08%)
miRNA (2.54%)
others (7.03%)
NONO-TFE3 interacting RNAs
G
Gene Expression Log2 (TPM+1)
p<2.22e-16
Non-overlapped
Overlapped
RNA-seq Log2 (Fold Change, dTAG13/DMSO)
p<2.17e-16
Non-overlapped
Overlapped
H
Flag-NONO-TFE3
Flag-TFE3 S321A
NaCl (mM) 10 50 100 150 200 300
MW (kDa)
Chromatin
α-Flag
α-CDK7
Soluble
α-Flag
α-CDK7
135
37
UOK109

Supplementary Figure 2. Identifying direct DNA and RNA binding targets of NONO-TFE3. (Related to Figure 1)

(A) Average metagene plots of CUT&Tag read intensities of NONO-TFE3 binding genes (TSS ± 5 kb) with or without dTAG13 (500 nM) at the indicated time points in UOK109 KI cells. n = 2 independent biological replicates.

(B) RIP-seq heatmaps of NONO-TFE3 binding genes from -2 kb TSS to +2 kb TES with or without dTAG13 (500 nM) treatment for 24 h in UOK109 KI cells. n = 2 independent biological replicates.

(C, D) Genomic Regions Enrichment of Annotations Tool (GREAT) and Gene Ontology (GO) analysis of NONO-TFE3 binding peaks identified from CUT&Tag (C) and RIP-seq (D), respectively.

(E) Motif enrichment of NONO-TFE3 bound regions identified by CUT&Tag analysis.

(F) Category annotation of NONO-TFE3 interacting RNAs identified from RIP-seq.

(G) Quantification of expression level (left) and fold changes after dTAG-13 treatment (right) of the genes exhibiting overlapped CUT&Tag and RIP-seq signals or CUT&Tag signals alone (non-overlapped). The box plots indicated the median (center line), the third and first quartiles (box limits) and 1.5x interquartile range (IQR) above and below the box (whiskers). (n = 2 independent biological replicates; two-sided Wilcoxon test). TPM, transcripts per kilobase million.

(H) Representative immunoblotting of chromatin bound and soluble fractions of NONO-TFE3 or NLS-TFE3-S321A at the indicated salt concentrations in transfected HEK 293T cells. CDK7 was used as an extraction loading control. n = 3 independent biological replicates. Source data are provided as a Source Data file

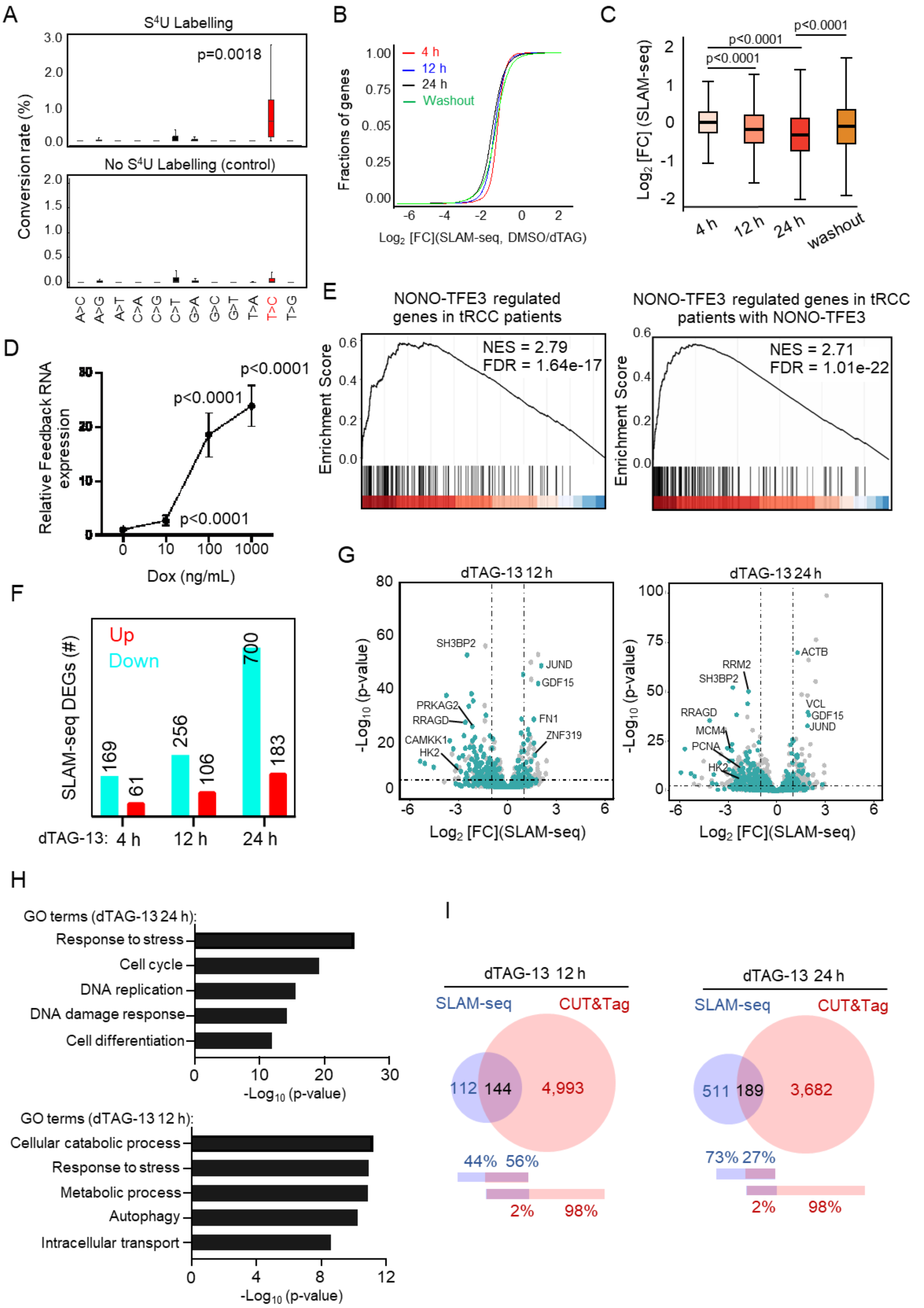
A
S4U Labelling
p=0.0018
No S4U Labelling (control)
Conversion rate (%)
A>C A>G A>T C>A C>G C>T G>A G>C G>T T>A T>C T>G
B
4 h
12 h
24 h
Washout
Fractions of genes
Log2 [FC](SLAM-seq, DMSO/dTAG)
C
p<0.0001
p<0.0001
p<0.0001
Log2 [FC] (SLAM-seq)
4 h
12 h
24 h
washout
D
Relative Feedback RNA expression
p<0.0001
p<0.0001
p<0.0001
Dox (ng/mL)
E
NONO-TFE3 regulated genes in tRCC patients
NES = 2.79
FDR = 1.64e-17
NONO-TFE3 regulated genes in tRCC patients with NONO-TFE3
NES = 2.71
FDR = 1.01e-22
Enrichment Score
F
Up
Down
SLAM-seq DEGs (#)
169
61
256
106
700
183
dTAG-13: 4 h 12 h 24 h
G
dTAG-13 12 h
SH3BP2
JUND
GDF15
PRKAG2
RRAGD
FN1
ZNF319
CAMKK1
HK2
dTAG-13 24 h
ACTB
RRM2
SH3BP2
RRAGD
VCL
GDF15
JUND
MCM4
PCNA
HK2
-Log10 (p-value)
Log2 [FC](SLAM-seq)
H
GO terms (dTAG-13 24 h):
Response to stress
Cell cycle
DNA replication
DNA damage response
Cell differentiation
-Log10 (p-value)
GO terms (dTAG-13 12 h):
Cellular catabolic process
Response to stress
Metabolic process
Autophagy
Intracellular transport
-Log10 (p-value)
I
dTAG-13 12 h
SLAM-seq
CUT&Tag
112
144
4,993
44% 56%
2% 98%
dTAG-13 24 h
SLAM-seq
CUT&Tag
511
189
3,682
73% 27%
2% 98%

Supplementary Figure 3. Identifying the primary transcriptional targets of NONO-TFE3 in tRCC. (related to Figure 2)

(A) Conversion rates for each base with or without S^4U labelling in SLAM-seq reads. Changes in the abundance of newly synthesized mRNAs (detected in SLAM-seq based on T to C (T>C) conversion). The box plots indicated the median (center line), the third and first quartiles (box limits) and 1.5x interquartile range (IQR) above and below the box (whiskers). (n = 2 independent biological replicates; two-sided Wilcoxon test; * $p < 0.05$).

(B) Transcription blocking index in dTAG-13 treated UOK109 KI cells at the indicated time points compared with the DMSO treated control group.

(C) The nascent transcriptional changes for genes across timepoints with NONO-TFE3 degradation kinetics. The box plots indicated the median (center line), the third and first quartiles (box limits) and 1.5x interquartile range (IQR) above and below the box (whiskers). (n = 2 independent biological replicates; two-sided Wilcoxon test).

(D) qRT-PCR analysis of relative RNA expression level (normalized to 0 ng/mL Dox) induced by indicated does of doxycycline (Dox). n = 6 independent biological replicates, one-way ANOVA with Tukey's post-hoc test. Data are shown as mean ± SD.

(E) Gene set enrichment analysis of NONO-TFE3 target genes identified from SLAM-seq in UOK109 KI cells and genes up-regulated (logFC > 3 and FDR < 0.05) in tRCC patients with various fusion partners (left, including NONO-TFE3 (3 cases), SFPQ-TFE3 (3 cases), ASPSCR1-TFE3 (8 cases), PRCC-TFE3 (6 cases), RBM10-TFE3 (1 case), MALTA1-TFEB (3 cases), CTCL1-TFEB (1 case), SFPQ-TFEB (1 case), ACTG1-MITF (1 case)) or NONO alone (right).

(F) Numbers (up- and down-regulated) of differentially expressed genes (DEGs) in UOK109 KI cells treated with dTAG 13 at the indicated time points.

(G) Volcano plot showing the genes differentially expressed after 12- or 24-hour dTAG-13 treatments.

(H) The Gene Ontology (GO) analysis of differential transcriptional genes identified from 12- or 24-h dTAG-13 treatment groups.

(I) Venn diagram showing the overlapped genes identified from time matched SLAM-seq and CUT&Tag analysis (comparison between before and 12- or 24-h after dTAG-13 treatment).

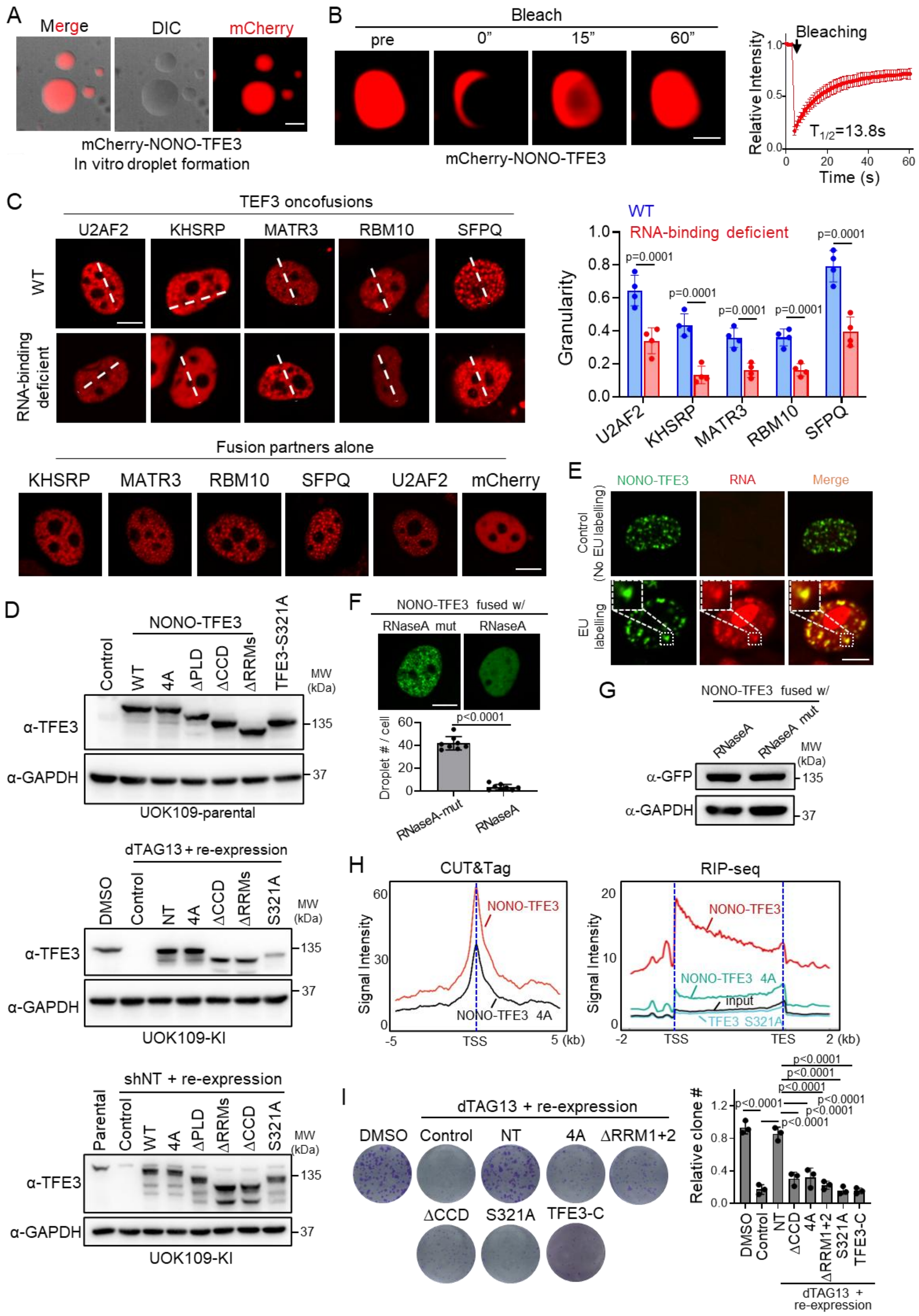
A
Merge
DIC
mCherry
mCherry-NONO-TFE3
In vitro droplet formation
B
Bleach
pre
0”
15”
60”
mCherry-NONO-TFE3
Bleaching
Relative Intensity
T1/2=13.8s
Time (s)
C
TEF3 oncofusions
U2AF2
KHSRP
MATR3
RBM10
SFPQ
WT
RNA-binding deficient
WT
RNA-binding deficient
Granularity
p=0.0001
Fusion partners alone
KHSRP
MATR3
RBM10
SFPQ
U2AF2
mCherry
D
NONO-TFE3
Control
WT
4A
ΔPLD
ΔCCD
ΔRRMs
TFE3-S321A
MW (kDa)
α-TFE3
α-GAPDH
UOK109-parental
dTAG13 + re-expression
DMSO
Control
NT
4A
ΔCCD
ΔRRMs
S321A
UOK109-KI
shNT + re-expression
Parental
Control
WT
4A
ΔPLD
ΔRRMs
ΔCCD
S321A
UOK109-KI
E
NONO-TFE3
RNA
Merge
Control (No EU labelling)
EU labelling
F
NONO-TFE3 fused w/
RNaseA mut
RNaseA
Droplet # / cell
p<0.0001
RNaseA-mut
RNaseA
G
NONO-TFE3 fused w/
RNaseA
RNaseA mut
MW (kDa)
α-GFP
α-GAPDH
H
CUT&Tag
Signal Intensity
NONO-TFE3
NONO-TFE3 4A
TSS
5 (kb)
RIP-seq
NONO-TFE3
NONO-TFE3 4A
Input
TFE3 S321A
TSS
TES
2 (kb)
I
dTAG13 + re-expression
DMSO
Control
NT
4A
ΔRRM1+2
ΔCCD
S321A
TFE3-C
Relative clone #
p<0.0001
DMSO
Control
NT
ΔCCD
4A
ΔRRM1+2
S321A
TFE3-C
dTAG13 + re-expression

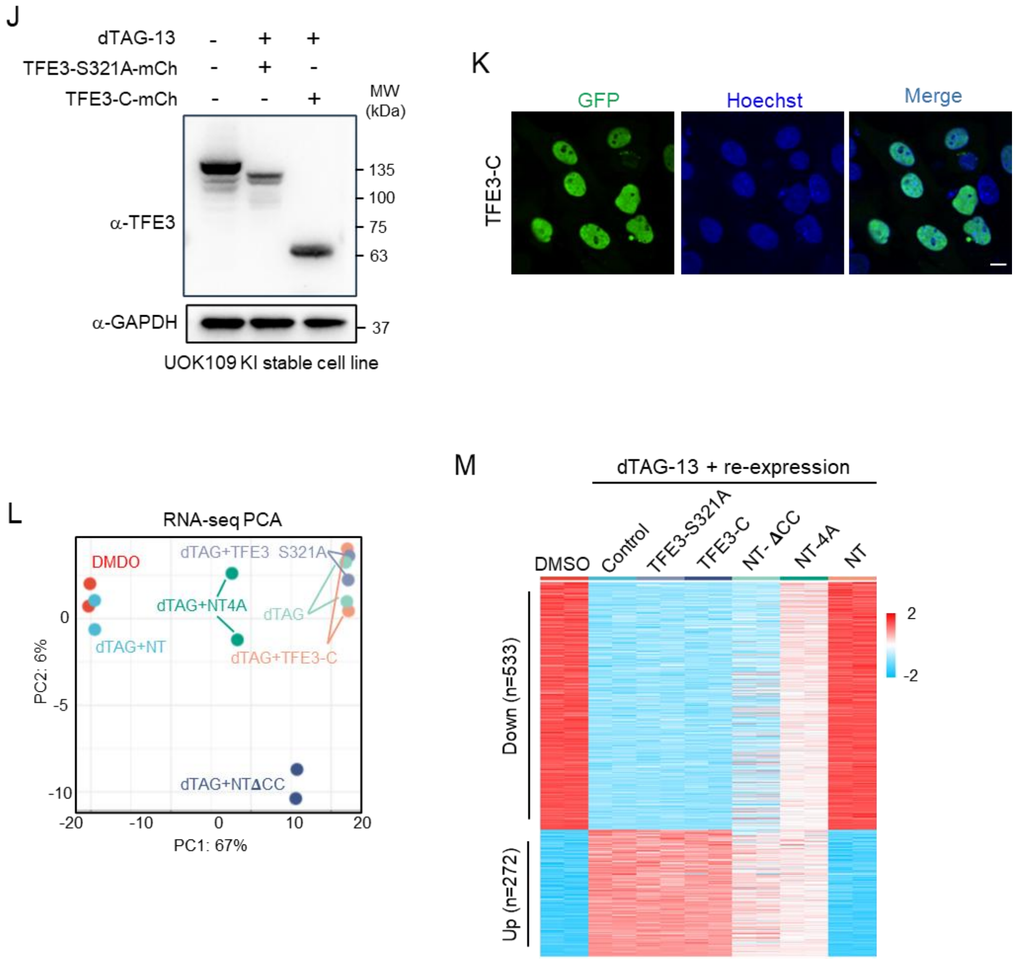
J
dTAG-13
TFE3-S321A-mCh
TFE3-C-mCh
MW (kDa)
α-TFE3
135
100
75
63
α-GAPDH
37
UOK109 KI stable cell line
K
GFP
Hoechst
Merge
TFE3-C
L
RNA-seq PCA
DMDO
dTAG+TFE3_S321A
dTAG+NT4A
dTAG
dTAG+NT
dTAG+TFE3-C
dTAG+NTΔCC
PC2: 6%
PC1: 67%
M
dTAG-13 + re-expression
DMSO
Control
TFE3-S321A
TFE3-C
NT- ΔCC
NT-4A
NT
Down (n=533)
Up (n=272)
2
-2

Supplementary Figure 4. RNA mediated condensation of TFE3 fusion proteins. (related to Figure 3)

(A) Representative fluorescence and differential interference contrast (DIC) images of the condensates of purified recombinant mCherry-fused NONO-TFE3. (n = 3 independent biological replicates), sale bar = 1 μm.

(B) Representative images (left) and normalized fluorescence signals (right) of purified recombinant mCherry fused NONO-TFE3 by FRAP assay. (n = 5 droplets from 3 independent biological replicates). The red arrow indicates the photobleaching starting time point. Scale bar = 1 μm.

(C) Representative images (left) and quantification (right) of droplet formation in HEK 293T cells transfected with the indicated TFE3 oncofusions in the presence or absence of RNA binding domains. Full length fusion partners alone were also included. Granularity, referring to the fluorescence signal fluctuation, is calculated as the standard deviation (S.D.) of the relative signal intensity along a line plot (normalized to the mean signal intensity of the entire line) at each pixel through an individual cell. (n = 8 cells from 3 independent biological replicates; one-way ANOVA with Tukey's post-hoc test). Scale bar, 10 μm. Data are shown as mean ± SD.

(D) Immunoblotting validation of the protein expression levels of NONO-TFE3 truncation variants in the parental UOK109 cells (top), UOK109 KI cells treated with dTAG13 (500 nM) (middle) and UOK109 KI cells expressing shRNA against NONO-TFE3 (bottom).

(E) Immunofluorescence of NONO-TFE3 and 5-ethynyl uridine (EU)-labelled RNA in UOK109 cells. Cells without EU in the culture medium were used as control. Scale bar, 10 μm.

(F) Representative images (top) and quantification (bottom) of the droplet numbers in UOK109 cells expressing NONO-TFE3 fused with wildtype RNase A and catalytically dead RNase A (RNase A mut). n = 8 cells from 3 independent biological replicates; two-sided unpaired Student's t-test). Scale bar, 10 μm. Data are shown as mean ± SD.

(G) Immunoblotting showing the equal expression of NONO-TFE3 fused with wildtype RNase A (RNase A-NT) and catalytically dead RNase A (RNase A mut). GAPDH was used as the loading control.

(H) Metagene analysis of CUT&Tag (left, centered on called WT peaks from -5 to 5 kb adjacent to TSS) and normalized RIP-seq intensities (right) of NONO-TFE3 binding genes from -2 kb TSS to +2 kb transcriptional end site (TES) for NONO-TFE3 WT and 4A mutant. TFE3 and input were also included in RIP-seq analysis as control.

(I) Representative images (left) and quantification (right) of colony formation assay results using UOK109 KI cells treated with dTAG13 (500 nM) and rescued with the expression of indicated NONO-TFE3 truncation variants. (n = 3 independent biological replicates; one-way ANOVA with Tukey's post-hoc test). Data are shown as mean ± SD.

(J) Immunoblotting showing the protein expression levels of NONO-TFE3, TFE3-S321A, and TFE3-C in dTAG-13-treated UOK109 KI stable cells.
(K) Immunofluorescence showing the nuclear localization of GFP fused TFE3-C. Hoechst was used for nuclear staining.
(L, M) PCA plot (L) and heatmap (M) of RNA-seq with dTAG-13 treated UOK109 KI cells stably expressing the indicated NONO-TFE3 truncations. NT: NONO-TFE3. n = 2 independent biological replicates. Source data are provided as a Source Data file.

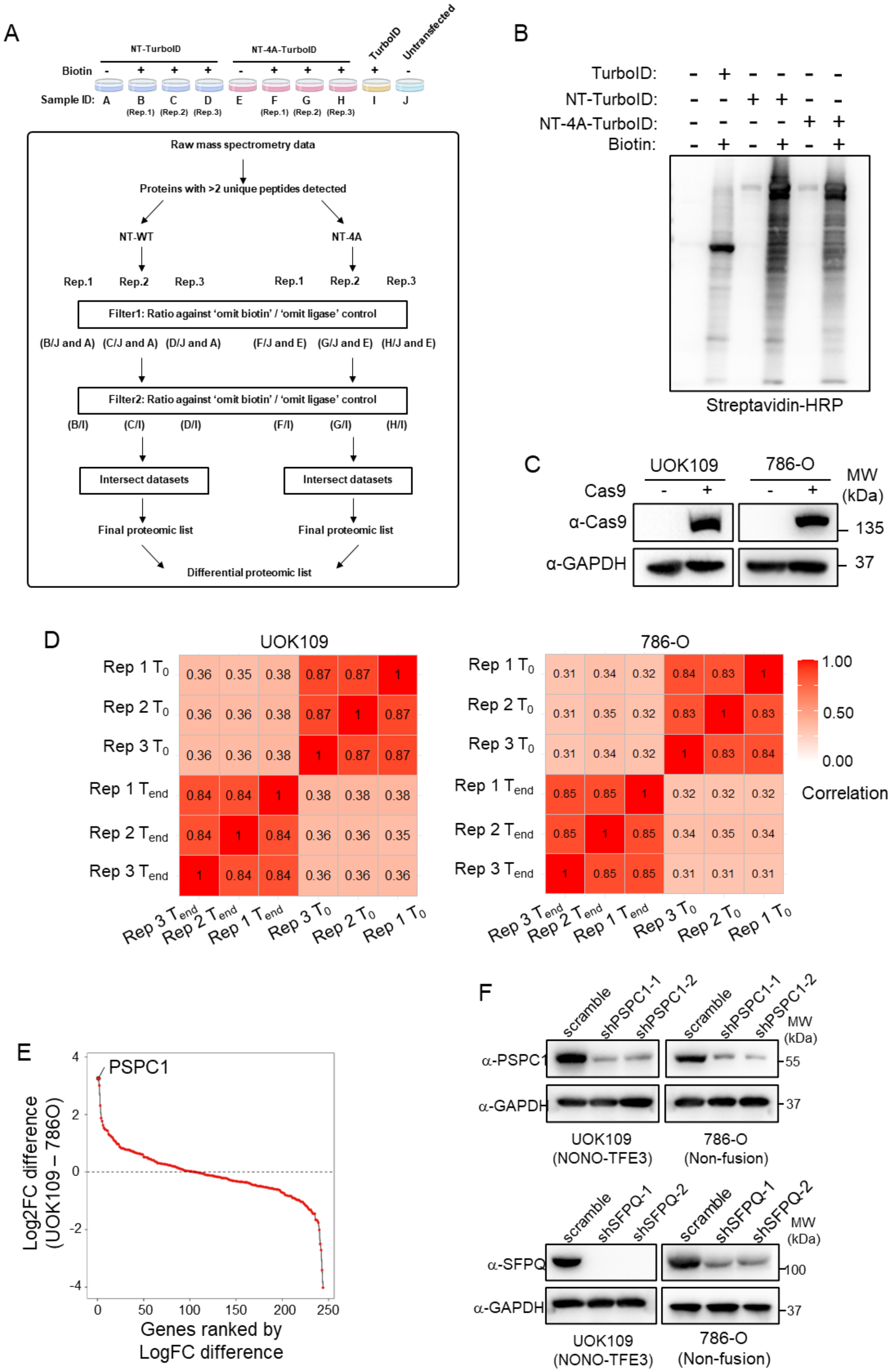
A
NT-TurboID
NT-4A-TurboID
TurboID
Untransfected
Biotin
Sample ID: A B C D E F G H I J
(Rep.1) (Rep.2) (Rep.3)
Raw mass spectrometry data
Proteins with >2 unique peptides detected
NT-WT
NT-4A
Rep.1 Rep.2 Rep.3
Filter1: Ratio against 'omit biotin' / 'omit ligase' control
(B/J and A) (C/J and A) (D/J and A) (F/J and E) (G/J and E) (H/J and E)
Filter2: Ratio against 'omit biotin' / 'omit ligase' control
(B/I) (C/I) (D/I) (F/I) (G/I) (H/I)
Intersect datasets
Final proteomic list
Differential proteomic list
B
TurboID:
NT-TurboID:
NT-4A-TurboID:
Biotin:
Streptavidin-HRP
C
UOK109
786-O
Cas9
α-Cas9
α-GAPDH
MW (kDa)
135
37
D
UOK109
786-O
Rep 1 T_0
Rep 2 T_0
Rep 3 T_0
Rep 1 T_{end}
Rep 2 T_{end}
Rep 3 T_{end}
1.00
0.50
0.00
Correlation
E
PSPC1
Log2FC difference (UOK109 – 786O)
Genes ranked by LogFC difference
F
scramble
shPSPC1-1
shPSPC1-2
α-PSPC1
α-GAPDH
55
37
UOK109 (NONO-TFE3)
786-O (Non-fusion)
shSFPQ-1
shSFPQ-2
α-SFPQ
100
MW (kDa)

Supplementary Figure 5. NONO-TFE3 co-condensates with PSPC1. (related to Figure 4)

(A) Schematic depicting the experimental setup and analytical procedures of TurboID proximity labelling with NONO-TFE3 and NONO-TFE3-4A.

(B) Immunoblotting analysis to confirm the biotinylation labelling with NONO-TFE3 and NONO-TFE3-4A fused to TurboID in UOK109 parental cells. (n = 3 independent biological replicates).

(C) Representative immunoblotting validation of UOK109 and 786-O cells stably expressing the Cas9 protein. These stable cell lines were used in subsequent CRISPR screening. (n = 3 independent biological replicates).

(D) Correlation analysis of three independent biological replicates of CRISPR screening at starting (T_0) or end (T_{end}) time points in UOK109 and 786-O cells.

(E) Fold-change (UOK109 /786-O) in sgRNA abundance in pooled RBP-focused CRISPR screening. Each dot represents the average of all sgRNAs targeting an RBP.

(F) Immunoblotting validation of PSPC1 and SFPQ knock-down in UOK109 and 786-O cells expressing the indicated shRNAs. (n = 3 independent biological replicates). Source data are provided as a Source Data file.

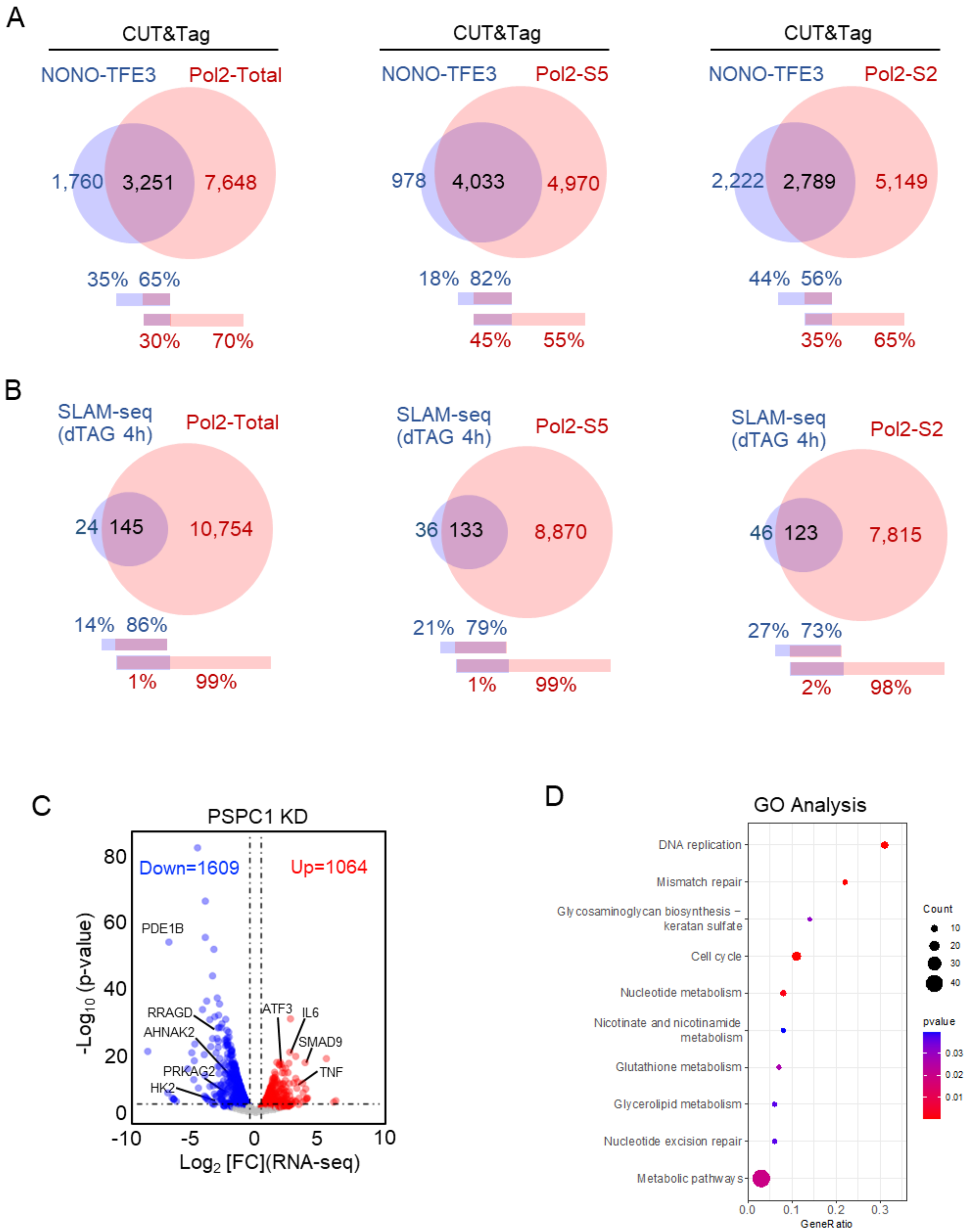

Supplementary Figure 6. NONO-TFE3 and PSPC1 co-condensation facilitates the recruitment of RNAPII. (related to Figure 5)

(A) Venn diagram showing the overlapped genes occupied by NONO-TFE3 and RNAPII-total (Pol II-total), RNAPII-Ser5p (Pol II-S5), or RNAPII-Ser2p (Pol II-S2), respectively.

(B) Venn diagram showing the overlapping of NONO-TFE3 target genes (SLAM-seq, 4-hour, **Figure 2**) and the occupancies of RNAPII-total (Pol II-total), RNAPII-Ser5p (Pol II-S5), or RNAPII-Ser2p (Pol II-S2), respectively.

(C) Volcano plot showing the genes differentially expressed (both up- and down-regulated) in UOK109 KI cells with PSPC1KD compared to the control group (scramble).

(D) The Gene Ontology (GO) analysis of differentially transcribed genes identified from **Supplementary Figure 5C.**

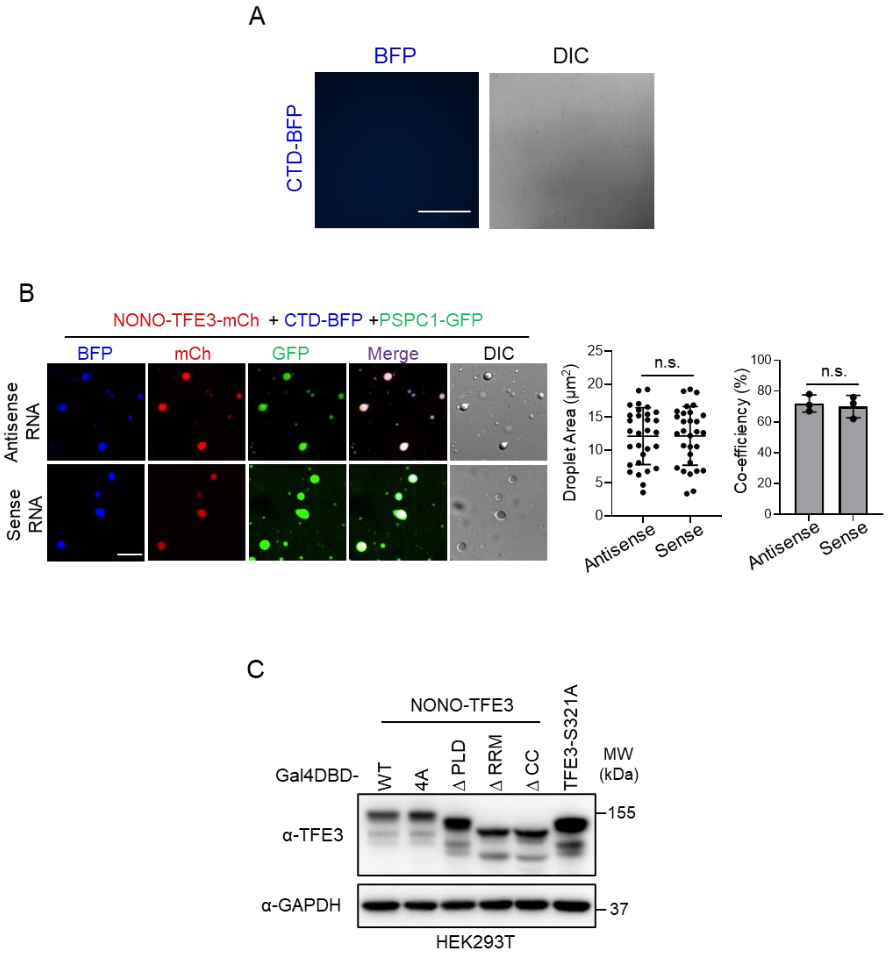

Supplementary Figure 7. NONO-TFE3 and PSPC1 co-condensation facilitates the recruitment of RNAPII. (related to Figures 6 and 7)

(A) In vitro droplet formation using recombinant BFP labelled RNAPII CTD proteins (CTD-BFP). Sale bar, 10 μm.

(B) Representative images (left) and quantification (right) of *in vitro* droplets formed by mCh-fused NONO-TFE3, BFP-fused Pol2 CTD and GFP-fused PSPC1 with antisense or sense strand RNA (200 bp from the transcription start site of the *RRM2* gene). Sale bar, 10 μm. (n = 30 droplets from 3 independent biological replicates; two-sided unpaired Student's t-test). Data are shown as mean ± SD.

(C) Immunoblotting analysis of the expression levels of Gal4DBD-fused NONO-TFE3 truncation variants or the TFE3 321A mutant in HEK293T cells. n = 3 independent biological replicates. Source d ata are provided as a Source Data file.

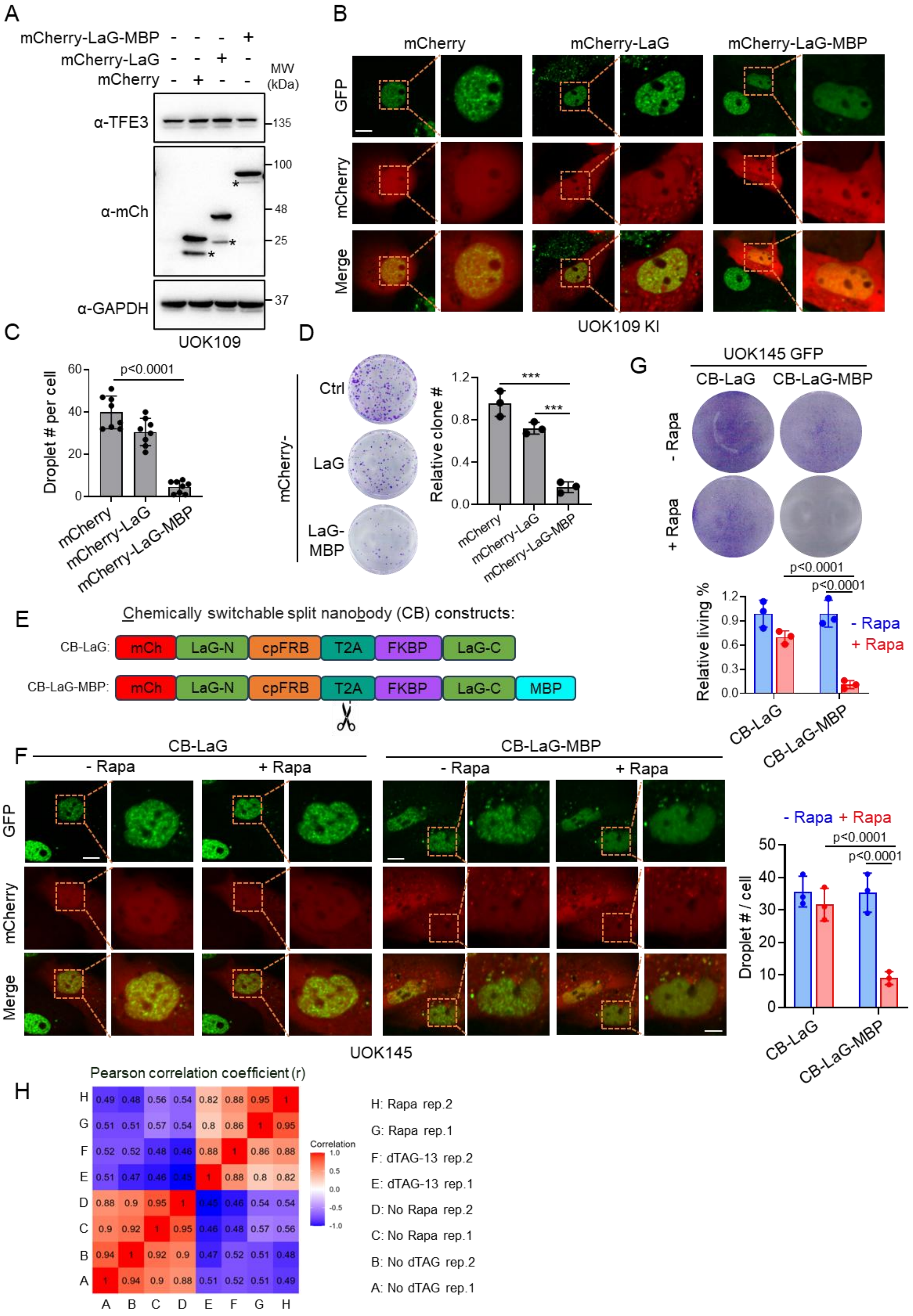
A
mCherry-LaG-MBP - - - +
mCherry-LaG - - + -
mCherry - + - -
MW (kDa)
α-TFE3
135
α-mCh
100
48
25
α-GAPDH
37
UOK109
B
mCherry
mCherry-LaG
mCherry-LaG-MBP
GFP
mCherry
Merge
UOK109 KI
C
Droplet # per cell
p<0.0001
60
40
20
0
mCherry
mCherry-LaG
mCherry-LaG-MBP
D
mCherry-
Ctrl
LaG
LaG-MBP
Relative clone #
1.2
0.8
0.4
0.0
***
***
mCherry
mCherry-LaG
mCherry-LaG-MBP
G
UOK145 GFP
CB-LaG
CB-LaG-MBP
- Rapa
+ Rapa
Relative living %
p<0.0001
p<0.0001
1.2
0.9
0.6
0.3
0.0
- Rapa
+ Rapa
CB-LaG
CB-LaG-MBP
E
Chemically switchable split nanobody (CB) constructs:
CB-LaG: mCh LaG-N cpFRB T2A FKBP LaG-C
CB-LaG-MBP: mCh LaG-N cpFRB T2A FKBP LaG-C MBP
F
CB-LaG
- Rapa
+ Rapa
CB-LaG-MBP
- Rapa
+ Rapa
GFP
mCherry
Merge
UOK145
- Rapa + Rapa
Droplet # / cell
p<0.0001
p<0.0001
50
40
30
20
10
0
CB-LaG
CB-LaG-MBP
H
Pearson correlation coefficient (r)
H 0.49 0.48 0.56 0.54 0.82 0.88 0.95 1
G 0.51 0.51 0.57 0.54 0.8 0.86 1 0.95
F 0.52 0.52 0.48 0.46 0.88 1 0.86 0.88
E 0.51 0.47 0.46 0.45 1 0.88 0.8 0.82
D 0.88 0.9 0.95 1 0.45 0.46 0.54 0.54
C 0.9 0.92 1 0.95 0.46 0.48 0.57 0.56
B 0.94 1 0.92 0.9 0.47 0.52 0.51 0.48
A 1 0.94 0.9 0.88 0.51 0.52 0.51 0.49
A B C D E F G H
Correlation
1.0
0.5
0.0
-0.5
-1.0
H: Rapa rep.2
G: Rapa rep.1
F: dTAG-13 rep.2
E: dTAG-13 rep.1
D: No Rapa rep.2
C: No Rapa rep.1
B: No dTAG rep.2
A: No dTAG rep.1

Supplementary Figure 8. Perturbation of TFE3 fuion protein condensation via chemogenetic manipulation. (related to Figure 8)

(A) Immunoblotting analysis of NONO-TFE3 protein expression levels in UOK109 cells transfected with the indicated plasmids. Asterisks (*) represent the non-specific bands of blotting with the mCherry (mCh) antibody. (n = 3 independent biological replicates).
(B, C) Representative images (B) and quantification (C) of droplet formation in UOK109 KI cells transfected with the indicated constructs. (n = 8 cells from 3 independent biological replicates; one-way ANOVA with Tukey's post-hoc test). Scale bar, 10 μm. Data are shown as mean ± SD.
(D) Representative images (left) and quantification (right) of clone formation assay results using UOK109 KI cells transfected with the indicated constructs. (n = 3 independent biological replicates; one-way ANOVA with Tukey's post-hoc test). Data are shown as mean ± SD.
(E) Schematic depicting the construct design for the chemically switchable split nanobody (CB) system.
(F) Representative images (left) and quantification (right) of droplet formation in UOK145 GFP cells (endogenous SFPQ-TFE3 replaced with GFP labeled SFPQ-TFE3 by knocking down SFPQ-TFE3 first and then re-expressing GFP-SFPQ-TFE3) transfected with the indicated constructs with or without rapamycin (Rapa). (n = 3 independent biological replicates; one-way ANOVA with Tukey's post-hoc test). Scale bar, 10 μm. Data are shown as mean ± SD.
(G) Representative images (top) and quantification (bottom) of clone formation assay results using UOK145 GFP cells as described in (F) with or without rapamycin (Rapa) treatment. (n = 3 independent biological replicates; one-way ANOVA with Tukey's post-hoc test). Data are shown as mean ± SD.
(H) Pearson correlation analysis of RNA-seq data from UOK109 KI cells treated with indicated conditions. n= 2 independent biological replicates. Source data are provided as a Source Data file.

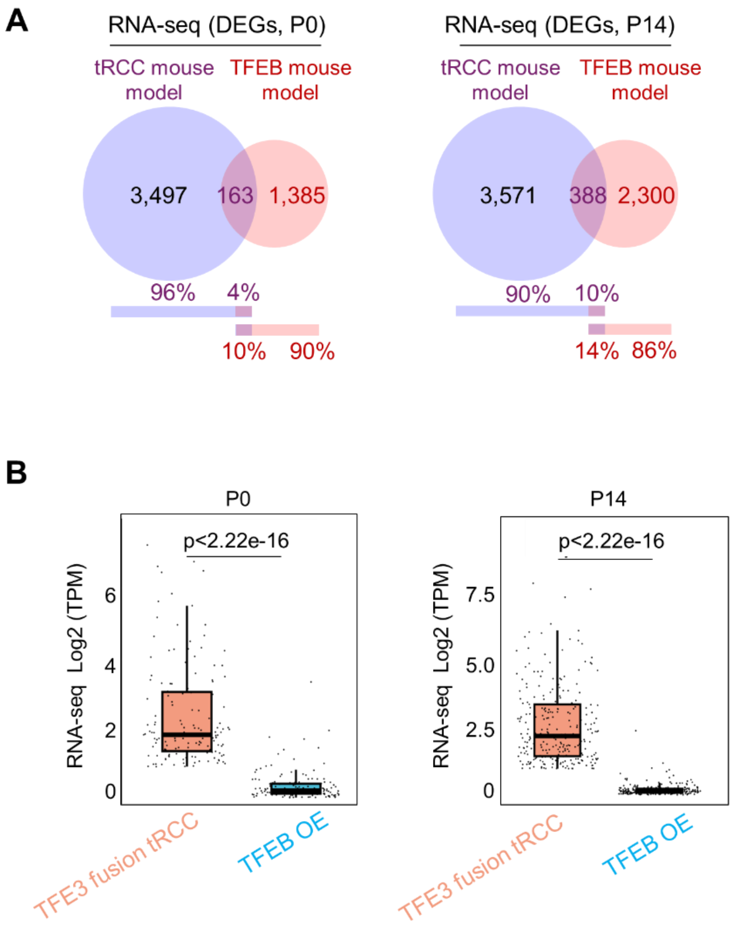

Supplementary Figure 9. Gene expression comparison between genetically modified mouse models expressing a TFE3 fusion gene (tRCC mouse model) or overexpressing TFEB (TFEB mouse model).

(A). Venn diagram showing the overlapping genes identified by RNA-seq in the indicated two mouse models at timepoint P0 and P14.

(B). Quantification of canonical TFEB/TFE3 targeted gene expression levels in the indicated mouse models. Box plots show the median (center line), the first and third quartiles (box limits), and the 1.5x interquartile range (IQR) above and below the box (whiskers; Two-sided Wilcoxon test). TPM, transcripts per kilobase million.
