# Supplementary material for: RNA-mediated condensation of TFE3 oncofusions facilitates transcriptional hub formation to promote translocation renal cell carcinoma
Source: Nat Commun. 2025 Sep 30;16:8712. doi: 10.1038/s41467-025-63761-z (PMC12484640; doi:10.1038/s41467-025-63761-z)
Supplement: Supplementary file 2 — Description of Additional Supplementary Files [file 41467_2025_63761_MOESM2_ESM.pdf]

Description of Additional Supplementary Files

File name: Supplementary Data 1

Description: CUT&Tag and RIP-seq peaks of NONO-TFE3 and Gene Ontology (GO) analysis (CUT&Tag) or Genomic Regions Enrichment of Annotations Tool (GREAT) analysis (RIP-seq) of identified peaks.

File name: Supplementary Data 2

Description: List and Gene Ontology (GO) analysis of Differentially Expressed Genes (DEGs) identified from SLAM-seq.

File name: Supplementary Data 3

Description: List of candidates identified from Turbo ID and CRISPR screening.

File name: Supplementary Data 4

Description: CUT&Tag peaks of RNAPII, PSPC1 and Differentially Expressed Genes (DEGs) identified from RNA-seq of PSPC1 knocking down UOK109 cells.

File name: Supplementary Data 5

Description: List of antibodies and primers.
