# Supplementary material for: RNA-mediated condensation of TFE3 oncofusions facilitates transcriptional hub formation to promote translocation renal cell carcinoma
Source: Nat Commun. 2025 Sep 30;16:8712. doi: 10.1038/s41467-025-63761-z (PMC12484640; doi:10.1038/s41467-025-63761-z)
Supplement: Supplementary file 8 — Reporting Summary [file 41467_2025_63761_MOESM8_ESM.pdf]

nature portfolio

Corresponding author(s): Yun Huang

Last updated by author(s): Aug 9, 2025

Reporting Summary

Nature Portfolio wishes to improve the reproducibility of the work that we publish. This form provides structure for consistency and transparency in reporting. For further information on Nature Portfolio policies, see our Editorial Policies and the Editorial Policy Checklist.

Statistics

For all statistical analyses, confirm that the following items are present in the figure legend, table legend, main text, or Methods section.

n/a	Confirmed	
☐	☒	The exact sample size (n) for each experimental group/condition, given as a discrete number and unit of measurement
☐	☒	A statement on whether measurements were taken from distinct samples or whether the same sample was measured repeatedly
☐	☒	The statistical test(s) used AND whether they are one- or two-sided<br>*Only common tests should be described solely by name; describe more complex techniques in the Methods section.*
☒	☐	A description of all covariates tested
☐	☒	A description of any assumptions or corrections, such as tests of normality and adjustment for multiple comparisons
☐	☒	A full description of the statistical parameters including central tendency (e.g. means) or other basic estimates (e.g. regression coefficient) AND variation (e.g. standard deviation) or associated estimates of uncertainty (e.g. confidence intervals)
☐	☒	For null hypothesis testing, the test statistic (e.g. F, t, r) with confidence intervals, effect sizes, degrees of freedom and P value noted<br>*Give P values as exact values whenever suitable.*
☒	☐	For Bayesian analysis, information on the choice of priors and Markov chain Monte Carlo settings
☒	☐	For hierarchical and complex designs, identification of the appropriate level for tests and full reporting of outcomes
☐	☒	Estimates of effect sizes (e.g. Cohen's d, Pearson's r), indicating how they were calculated

Our web collection on statistics for biologists contains articles on many of the points above.

Software and code

Policy information about availability of computer code

Data collection	The fluorescence imaging data were obtained by using a Nikon NIS-Elements AR Confocal Microscope.<br>Western blot results were obtained using a ChemiDoc Imaging system (Bio-Rad).<br>Sequencing data are acquired with the following platforms:<br>Hiseq 3000 (illumina)<br>NextSeq500 (illumina)<br>No custom codes were used.
Data analysis	ImageJ V1.53 (NIH), GraphPad Prism 9<br><br>All the software and code used for data analysis are open source and publically available. Detailed sequencing data analysis are listed in Materials and Methods section.

For manuscripts utilizing custom algorithms or software that are central to the research but not yet described in published literature, software must be made available to editors and reviewers. We strongly encourage code deposition in a community repository (e.g. GitHub). See the Nature Portfolio guidelines for submitting code & software for further information.

Data

Policy information about availability of data

All manuscripts must include a data availability statement. This statement should provide the following information, where applicable:

- Accession codes, unique identifiers, or web links for publicly available datasets
- A description of any restrictions on data availability
- For clinical datasets or third party data, please ensure that the statement adheres to our policy

The sequencing datasets were deposited into the NCBI BioProject under the accession number PRJNA1129016(https://dataview.ncbi.nlm.nih.gov/object/PRJNA1129016?reviewer=td1ujtse7efbksbqh3bss86bf)

Research involving human participants, their data, or biological material

Policy information about studies with human participants or human data. See also policy information about sex, gender (identity/presentation), and sexual orientation and race, ethnicity and racism.

Reporting on sex and gender	our study did not involve human research participants
Reporting on race, ethnicity, or other socially relevant groupings	our study did not involve human research participants
Population characteristics	our study did not involve human research participants
Recruitment	our study did not involve human research participants
Ethics oversight	our study did not involve human research participants

Note that full information on the approval of the study protocol must also be provided in the manuscript.

Field-specific reporting

Please select the one below that is the best fit for your research. If you are not sure, read the appropriate sections before making your selection.

☒ Life sciences ☐ Behavioural & social sciences ☐ Ecological, evolutionary & environmental sciences

For a reference copy of the document with all sections, see nature.com/documents/nr-reporting-summary-flat.pdf

Life sciences study design

All studies must disclose on these points even when the disclosure is negative.

Sample size	No statistic method was used to predetermine sample size. The sample sizes in our experiments were determined based on previous experience and standards in the field (PMID:37591951) and were determined to ensure significance in statistic tests, or harvest enough sorted cells for the molecular experiments and high throughput sequencing. For the mice phenotype analysis, at least 3 mice for each group were carried out.The sample size is sufficient since we could detect large difference between two experimental conditions, with P-value lower than 0.05.
Data exclusions	No samples were excluded.
Replication	All experiments were reproduced reliably in independent settings for at least two (RNA-seq, SLAM- seq, Cut&Tag) or more than three times (Imaging, molecular or cell biology experiments). The replicates number is reported in the figure legend.
Randomization	Samples were allocated into experimental groups through genotype differences. Randomization was not considered in this study and all the samples were treated and analyzed in the same manner.
Blinding	Blinding was not considered in this study as knowledge of sample identity was required to perform analysis and comparisons.

Reporting for specific materials, systems and methods

We require information from authors about some types of materials, experimental systems and methods used in many studies. Here, indicate whether each material, system or method listed is relevant to your study. If you are not sure if a list item applies to your research, read the appropriate section before selecting a response.

Materials & experimental systems

n/a	Involved in the study
☐	☒ Antibodies
☐	☒ Eukaryotic cell lines
☒	☐ Palaeontology and archaeology
☐	☒ Animals and other organisms
☒	☐ Clinical data
☒	☐ Dual use research of concern
☒	☐ Plants

Methods

n/a	Involved in the study
☐	☒ ChIP-seq
☒	☐ Flow cytometry
☒	☐ MRI-based neuroimaging

Antibodies

Antibodies used	GFP Santa Cruz Biotechnology sc-9996. (1:1000 dilution)<br>mCherry NOVUS NBP2-25157.(1:1000 dilution)<br>TFE3 Cell Signaling Technology 14779S.(1:1000 dilution)<br>GAPDH Santa Cruz Biotechnology sc-32233.(1:2000 dilution)<br>FLAG Sigma-Aldrich F1804.(1:5000 dilution for blotting,1:100 dilution for IP and CUT&Tag)<br>CDK7 Santa Cruz Biotechnology sc-7344.(1:1000 dilution)<br>Streptavidin-HRP Cell signaling technology 3999.(1:1000 dilution)<br>PSPC1 Santa Cruz Biotechnology sc-374181.(1:1000 dilution)<br>SFPQ Abcam ab177149.(1:1000 dilution)<br>IgG NOVUS IG266.(1:100 dilution)<br>Cas9 Santa Cruz Biotechnology sc-517386.(1:1000 dilution)<br>FKBP12 Thermo Scientific PA1026A.(1:1000 dilution)<br>Total Pol-II Abcam ab26721.(1:100 dilution)<br>Pol-II Ser2 Active Motif 61984.(1:100 dilution)<br>Pol-II Ser5 Abcam ab5131.(1:100 dilution)<br>H3K27ac Diagenode C15410196.(1:100 dilution)<br>Anti-Rabbit Secondary Antibody EpiCypher 13-0047.(1:100 dilution)<br>Anti-Mouse Secondary Antibody EpiCypher 13-0048.(1:100 dilution)
Validation	All commercial antibodies were commerciallly available and validated on the manufacturers and in previous publications.

Eukaryotic cell lines

Policy information about cell lines and Sex and Gender in Research

Cell line source(s)	HEK293T (CRL-3216)and 786-O (CRL-1932) cells were purchased from ATCC. UOK109, UOK145 and UOK146 cells were kindly provided by Dr. W. Marston Linehan (National Cancer Institute). U2OS cells with Lac operators were kindly provided by Dr. David Spector (Cold Spring Harbor Laboratory).
Authentication	The authentication of HEK293T and 786-O cells were performed by commercial providers using morphology. UOK109, UOK145 and UOK146 cells were confirmed by Sanger sequencing at the TFE3 fusion sites. U2OS cells were confirmed by imaging with LacO staining. Single clone of UOK109 KI was selected and further confirmed by sequencing the genomic DNA of the knockin junction site at the C-terminus of NONO-TFE3 and immunoblot with Flag and GFP antibody.
Mycoplasma contamination	Mycoplasma contamination was tested by using the LookOut PCR detection kit from SigmaAldrich. All cell lines used were microplasma free.
Commonly misidentified lines<br>(See ICLAC register)	No commonly misidentified cell lines were used.

Animals and other research organisms

Policy information about studies involving animals; ARRIVE guidelines recommended for reporting animal research, and Sex and Gender in Research

Laboratory animals	C.B-Igh-1b/GbmsTac-Prkdcscid-Lystbg N7 mice (SCID-Beige) from Taconic Biosciences. 8-12-week-old female and mice were used for human tRCC cell transplantation experiments.<br><br>All animals-related studies were maintained in the animal facility of Texas A&M University Institute of Biosciences and Technology and approved by the Institutional Animal Care and Use Committee (IACUC). Mice were kept under light-dark cycles of 12h:12h in a climate-controlled environment.
Wild animals	No wild animals were used in the study
Reporting on sex	Female and male mice are both chosen with no bais

Field-collected samples	There were no samples collected in the field
Ethics oversight	Animal experiments in this study were approved and carried out in accordance with the protocol provided by the Institutional Animal Care and Use Committee (IACUC) at the Institute of Biosciences and Technology, School of Medicine, Texas A&M University.

Note that full information on the approval of the study protocol must also be provided in the manuscript.

Plants

Seed stocks	N/A
Novel plant genotypes	N/A
Authentication	N/A

ChIP-seq

Data deposition

☒ Confirm that both raw and final processed data have been deposited in a public database such as GEO.

☒ Confirm that you have deposited or provided access to graph files (e.g. BED files) for the called peaks.

Data access links
May remain private before publication.

https://dataview.ncbi.nlm.nih.gov/object/PRJNA1129016?reviewer=td1ujtse7efbksbqh3bss86bf

Files in database submission

NONO-TFE3 CUT&Tag
SRR29608235 UOK109_CUT_dTAG0h_flag_rep1
SRR29608234 UOK109_CUT_dTAG0h_flag_rep2
SRR29608223 UOK109_CUT_dTAG4h_flag_rep1
SRR29608212 UOK109_CUT_dTAG4h_flag_rep2
SRR29608214 UOK109_CUT_76wt_flag_rep1
SRR29608213 UOK109_CUT_76wt_flag_rep2
SRR29608201 UOK109_CUT_dTAG12h_flag_rep1
SRR29608190 UOK109_CUT_dTAG12h_flag_rep2
SRR29608179 UOK109_CUT_dTAG24h_flag_rep1
SRR29608168 UOK109_CUT_dTAG24h_flag_rep2
SRR29608166 UOK109_CUT_washout_flag_rep1
SRR29608165 UOK109_CUT_washout_flag_rep2

TFE3 CUT&Tag
SRR29608211 UOK109_CUT_TFE3SA_flag_rep1
SRR29608210 UOK109_CUT_TFE3SA_flag_rep2

total Pol2 CUT&Tag
SRR29608220 UOK109_CUT_shcontrol_tp2_rep1
SRR29608219 UOK109_CUT_shcontrol_tp2_rep2
SRR29608218 UOK109_CUT_shPSPC1_tp2_rep1
SRR29608217 UOK109_CUT_shPSPC1_tp2_rep2
SRR29608233 UOK109_CUT_dTAG0h_tp2_rep1
SRR29608232 UOK109_CUT_dTAG0h_tp2_rep2
SRR29608231 UOK109_CUT_dTAG4h_tp2_rep1
SRR29608230 UOK109_CUT_dTAG4h_tp2_rep2

Ser5 CUT&Tag
SRR29608229 UOK109_CUT_dTAG0h_ser5_rep1
SRR29608228 UOK109_CUT_dTAG0h_ser5_rep2
SRR29608227 UOK109_CUT_dTAG4h_ser5_rep1
SRR29608226 UOK109_CUT_dTAG4h_ser5_rep2

Ser2 CUT&Tag
SRR29608225 UOK109_CUT_dTAG0h_ser2_rep1
SRR29608224 UOK109_CUT_dTAG0h_ser2_rep2
SRR29608222 UOK109_CUT_dTAG4h_ser2_rep1
SRR29608221 UOK109_CUT_dTAG4h_ser2_rep2

PSPC1 CUT&Tag

SRR29608216 UOK109_CUT_PSPC1_rep1
SRR29608215 UOK109_CUT_PSPC1_rep2

H3K27ac CUT&Tag
SRR30882587 UOK109_CUT_H3K27ac

H3K4me3 CUT&Tag
SRR30882586 UOK109_CUT_H3K4me3_rep1
SRR30882585 UOK109_CUT_H3K4me3_rep2

Genome browser session (e.g. UCSC)

As requested by our university, the server cannot be accessed by public resources such as UCSC genome browser. We will provide the link upon request.

Methodology

Replicates

we had 2 replicates in each experimental group

Sequencing depth

NONO-TFE3 CUT&Tag
SRR29608235 UOK109_CUT_dTAG0h_flag_rep1 CoveredSite: 682181157 Coverage(%): 22.09 MeanDepth: 0.96
SRR29608234 UOK109_CUT_dTAG0h_flag_rep2 CoveredSite: 675582189 Coverage(%): 21.88 MeanDepth: 0.98
SRR29608223 UOK109_CUT_dTAG4h_flag_rep1 CoveredSite: 758464067 Coverage(%): 24.56 MeanDepth: 0.54
SRR29608212 UOK109_CUT_dTAG4h_flag_rep2 CoveredSite: 828356931 Coverage(%): 26.82 MeanDepth: 0.61
SRR29608214 UOK109_CUT_76wt_flag_rep1 CoveredSite: 534093257 Coverage(%): 17.29 MeanDepth: 1.21
SRR29608213 UOK109_CUT_76wt_flag_rep2 CoveredSite: 569620472 Coverage(%): 18.44 MeanDepth: 1.31
SRR29608201 UOK109_CUT_dTAG12h_flag_rep1 CoveredSite: 634957122 Coverage(%): 20.56 MeanDepth: 0.42
SRR29608190 UOK109_CUT_dTAG12h_flag_rep2 CoveredSite: 616145459 Coverage(%): 19.95 MeanDepth: 0.40
SRR29608179 UOK109_CUT_dTAG24h_flag_rep1 CoveredSite: 298079467 Coverage(%): 9.65 MeanDepth: 0.17
SRR29608168 UOK109_CUT_dTAG24h_flag_rep2 CoveredSite: 470927033 Coverage(%): 15.25 MeanDepth: 0.32
SRR29608166 UOK109_CUT_washout_flag_rep1 CoveredSite: 638856099 Coverage(%): 20.69 MeanDepth: 0.66
SRR29608165 UOK109_CUT_washout_flag_rep2 CoveredSite: 785383551 Coverage(%): 25.43 MeanDepth: 0.88

TFE3 CUT&Tag
SRR29608211 UOK109_CUT_TFE3SA_flag_rep1 CoveredSite: 407998797 Coverage(%): 13.21 MeanDepth: 0.49
SRR29608210 UOK109_CUT_TFE3SA_flag_rep2 CoveredSite: 435459091 Coverage(%): 14.10 MeanDepth: 0.53

total Pol2 CUT&Tag
SRR29608220 UOK109_CUT_shcontrol_tp2_rep1 CoveredSite: 721736243 Coverage(%): 23.37 MeanDepth: 1.04
SRR29608219 UOK109_CUT_shcontrol_tp2_rep2 CoveredSite: 656355271 Coverage(%): 21.25 MeanDepth: 0.88
SRR29608218 UOK109_CUT_shPSPC1_tp2_rep1 CoveredSite: 664307689 Coverage(%): 21.51 MeanDepth: 0.94
SRR29608217 UOK109_CUT_shPSPC1_tp2_rep2 CoveredSite: 649450644 Coverage(%): 21.03 MeanDepth: 0.90
SRR29608233 UOK109_CUT_dTAG0h_tp2_rep1 CoveredSite: 760126552 Coverage(%): 24.61 MeanDepth: 1.10
SRR29608232 UOK109_CUT_dTAG0h_tp2_rep2 CoveredSite: 792405682 Coverage(%): 25.66 MeanDepth: 1.20
SRR29608231 UOK109_CUT_dTAG4h_tp2_rep1 CoveredSite: 651012746 Coverage(%): 21.08 MeanDepth: 0.93
SRR29608230 UOK109_CUT_dTAG4h_tp2_rep2 CoveredSite: 714280178 Coverage(%): 23.13 MeanDepth: 1.12

Ser5 CUT&Tag
SRR29608229 UOK109_CUT_dTAG0h_ser5_rep1 CoveredSite: 891157746 Coverage(%): 28.86 MeanDepth: 1.26
SRR29608228 UOK109_CUT_dTAG0h_ser5_rep2 CoveredSite: 922810375 Coverage(%): 29.88 MeanDepth: 1.36
SRR29608227 UOK109_CUT_dTAG4h_ser5_rep1 CoveredSite: 933095855 Coverage(%): 30.21 MeanDepth: 1.20
SRR29608226 UOK109_CUT_dTAG4h_ser5_rep2 CoveredSite: 1066447451 Coverage(%): 34.53 MeanDepth: 1.53

Ser2 CUT&Tag
SRR29608225 UOK109_CUT_dTAG0h_ser2_rep1 CoveredSite: 729464126 Coverage(%): 23.62 MeanDepth: 0.91
SRR29608224 UOK109_CUT_dTAG0h_ser2_rep2 CoveredSite: 729464126 Coverage(%): 23.62 MeanDepth: 0.91
SRR29608222 UOK109_CUT_dTAG4h_ser2_rep1 CoveredSite: 765653093 Coverage(%): 24.79 MeanDepth: 1.03
SRR29608221 UOK109_CUT_dTAG4h_ser2_rep2 CoveredSite: 877638162 Coverage(%): 28.42 MeanDepth: 1.33

PSPC1 CUT&Tag
SRR29608216 UOK109_CUT_PSPC1_rep1 CoveredSite: 469345546 Coverage(%): 15.20 MeanDepth: 0.94
SRR29608215 UOK109_CUT_PSPC1_rep2 CoveredSite: 462288047 Coverage(%): 14.97 MeanDepth: 0.93

Antibodies

FLAG Sigma-Aldrich F1804
Total Pol-II Abcam ab26721
Pol-II Ser2 Active Motif 61984
Pol-II Ser5 Abcam ab5131
H3K27ac Diagenode C15410196
Anti-Rabbit Secondary Antibody EpiCypher 13-0047
Anti-Mouse Secondary Antibody EpiCypher 13-0048

Peak calling parameters

default parameters

Data quality

FastQC v0.12.1 is used to check the sequencing quality.

For RNA-seq, differentially expressed genes were identified using DESeq2 package (v1.38.3) [PMID: 25516281] with the criteria of |log2FC|>1 and FDR<0.05. Raw bam files were converted to bigwig format using bamCoverage (v3.5.4) [PMID: 27079975] with "--normalizeUsing RPKM" to visualize in Integrated Genome Viewer (IGV, v2.13.2)

For CUT&Tag, PCR duplicates were marked and removed using picard pipeline (v2.27.4, https://broadinstitute.github.io/picard/). Peak calling was conducted using MACS2 (v2.2.9.1) [PMID: 18798982] with default narrow or broad calling parameters (q-value<0.001), and peaks located within a distance of 5 kb around transcription start site were defined as promoter peaks. Reads on the peaks were counted using bedtools (v2.31.1) [PMID: 20110278] and converted to reads per kilobase per million mapped reads (RPKM). CUT&Tag binding signals were normalized using deepTools bamCoverage (v3.5.4) [PMID: 27079975] with murine spike-in supplied by kit for comparing samples, and visualized using deepTools computeMatrix (v3.5.4) [PMID: 27079975] and Integrated Genome Viewer (IGV, v2.13.2) [PMID: 22517427].

For SLAM-seq, quantification and differentially expressed analysis of nascent RNA were performed with GRAND-SLAM (v2.0.7b) [PMID: 29949974] and grandR (v0.2.5) pipeline [PMID: 37321987]. Genes with |log2FC|>1 and FDR<0.05 were statistically significant

For RIP-seq, PCR duplicates were marked and removed using picard pipeline (v2.27.4, https://broadinstitute.github.io/picard/). Alignments on "+" and "-" strands were filtered separately to identify strand-specific peaks using MACS2 (v2.2.9.1) [PMID: 18798982] with default parameters (q-value<0.001), and peaks located within a distance of 5 kb around transcription start site were defined as promoter peaks. Reads on the peaks were counted using the summarizeOverlaps function from GenomicAlignments (v1.34.1) [PMID: 23950696] package and converted to reads per kilobase per million mapped reads (RPKM). RIP binding signals were RPKM-normalized using deepTools bamCoverage (v3.5.4) [PMID: 27079975], and visualized using deepTools computeMatrix (v3.5.4) [PMID: 27079975] and Integrated Genome Viewer (IGV, v2.13.2) [PMID: 22517427]..

Software

FastQC (v0.12.1), bowtie2 (v 2.5.1), samtools (v1.6), picard (v2.27.4), MACS2 (v2.2.9.1), bedtools (v2.31.1), deepTools bamCoverage (v3.5.4), deepTools computeMatrix (v3.5.4), Integrated Genome Viewer (v2.13.2)
